# Dopamine and the Gut Microbiota: Interactions Within the Microbiota–Gut–Brain Axis and Therapeutic Perspectives

**DOI:** 10.3390/ijms27010271

**Published:** 2025-12-26

**Authors:** Aurelia Cristiana Barbu, Smaranda Stoleru, Aurelian Zugravu, Elena Poenaru, Adrian Dragomir, Mihnea Costescu, Sorina Maria Aurelian, Yara Shhab, Clara Maria Stoleru, Oana Andreia Coman, Ion Fulga

**Affiliations:** 1 Faculty of Medicine, “Carol Davila” University of Medicine and Pharmacy, 050474 Bucharest, Romania; aurelia-cristiana.barbu@drd.umfcd.ro (A.C.B.); smaranda.stoleru@umfcd.ro (S.S.); oana.coman@umfcd.ro (O.A.C.);; 2 Faculty of Medical Engineering, National University of Science and Technology Politehnica Bucharest, 060042 Bucharest, Romania; clara.stoleru@gmail.com

**Keywords:** dopamine, gut microbiota, microbiota–gut–brain axis, Parkinson’s disease, L-DOPA, neurogastroenterology

## Abstract

The microbiota–gut–brain axis (MGBA) comprises a complex bidirectional communication network integrating neural, immune, metabolic, and endocrine pathways. Dopamine, traditionally viewed as a central neurotransmitter, also plays essential roles in the gastrointestinal (GI) tract, where it regulates motility, secretion, barrier homeostasis, and mucosal immunity. Growing evidence indicates that the gut microbiota significantly contributes to intestinal dopamine metabolism through specialized enzymatic pathways, particularly tyrosine decarboxylase in *Enterococcus* species and catechol dehydroxylase in *Eggerthella* species. These microbial reactions compete with host processes, alter dopaminergic tone, and degrade orally administered levodopa (L-DOPA), providing a mechanistic explanation for the variability in treatment response in Parkinson’s disease (PD). Beyond PD, microbially mediated alterations in dopaminergic signaling have been implicated in mood disorders, neurodevelopmental conditions, metabolic dysfunction, and immune-mediated diseases. This review synthesizes current mechanistic and translational evidence on the dopamine–microbiota interface, outlines microbial pathways shaping dopaminergic activity, and highlights therapeutic opportunities including microbiota modulation, dietary strategies, fecal microbiota transplantation, and targeted inhibitors of microbial dopamine metabolism. Understanding this interface offers a foundation for developing personalized approaches in neurogastroenterology and neuromodulatory therapies.

## 1. Introduction

The microbiota–gut–brain axis (MGBA) is a key framework for understanding how intestinal microbes influence neural, immune, and metabolic pathways that shape brain function. Among its molecular mediators, neurotransmitters have a central role. While serotonin and GABA have been extensively studied, dopamine remains comparatively underexplored despite its relevance to reward, motivation, and motor control [1,2,3].

A substantial fraction of peripheral dopamine is generated in the gastrointestinal tract. Enterochromaffin cells and enteric neurons synthesize dopamine locally, regulating motility, epithelial barrier function, secretion, and immune responses. Although peripheral dopamine does not cross the blood–brain barrier, it can influence central activity indirectly through vagal, immune, endocrine, and metabolic routes, positioning the gut as an important dopaminergic hub [4,5].

A major conceptual advance is the recognition that gut microbes harbor enzymatic pathways that metabolize dopamine and its precursor L-DOPA [6,7]. In particular, bacterial tyrosine decarboxylase (TyrDC) in *Enterococcus* and catechol dehydroxylase (Dadh) in *Eggerthella* can convert L-DOPA to dopamine and subsequently to *m*-tyramine. These pathways are not inhibited by carbidopa and can alter L-DOPA pharmacokinetics [8,9,10].

Parkinson’s disease (PD) provides the clearest clinical context. PD-associated dysbiosis, including enrichment of *Enterococcus* and *Eggerthella*, correlates with gastrointestinal dysfunction and variable L-DOPA response [11,12,13]. However, microbial dopamine metabolism may also be relevant to psychiatric, neurodevelopmental, metabolic, and immune-mediated disorders [14,15,16].

This review integrates mechanistic, preclinical, and clinical evidence on dopamine–microbiota interactions, outlines MGBA communication routes, and summarizes emerging microbiome-informed therapeutic strategies.

## 2. Methods

A narrative literature review was conducted using PubMed, Scopus, and Web of Science with the following terms: “dopamine”, “gut microbiota”, “L-DOPA”, “Parkinson’s disease”, “tyrosine decarboxylase”, and “microbiota–gut–brain axis”. Articles published between 1995 and 2025 were considered. The search yielded 420 records; after deduplication and screening, 100 full-text articles were assessed.

Original research articles, clinical studies, systematic reviews, and major narrative reviews were included. Non-English articles, conference abstracts without primary data, and case reports without mechanistic relevance were excluded. Approximately 20 core mechanistic studies were prioritized based on mechanistic validity, clarity, and translational relevance. Two authors independently screened references. No quantitative meta-analysis was performed due to heterogeneity in experimental models and endpoints.

## 3. Dopamine in the Gastrointestinal Tract

Dopamine is a key peripheral signaling molecule in the gastrointestinal tract, modulating motility, secretion, epithelial barrier integrity, and mucosal immune responses. Endogenous intestinal dopamine is primarily produced by enterochromaffin cells and enteric neurons (Table 1). Acting through D1-like and D2-like receptors expressed on smooth muscle, epithelial, enteric neuronal, and immune cells, dopamine regulates peristalsis, fluid–electrolyte transport, and barrier homeostasis within the MGBA context [7,14].

**Figure 1:**
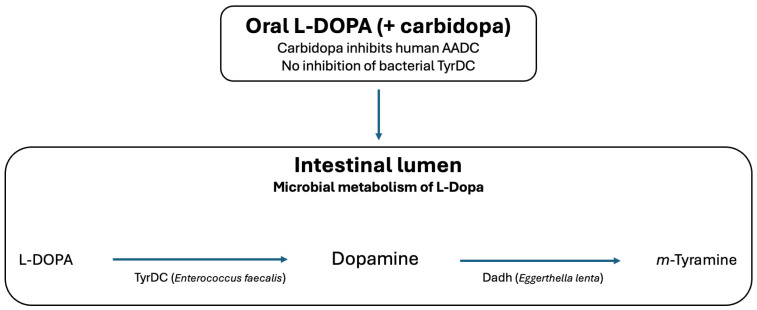
Microbial metabolism of orally administered L-DOPA in the intestinal lumen. Although carbidopa inhibits human aromatic L-amino acid decarboxylase (AADC), it does not inhibit bacterial tyrosine decarboxylase (TyrDC). Consequently, gut bacteria such as *Enterococcus faecalis* convert L-DOPA to dopamine, which can be further metabolized by *Eggerthella lenta* via dopamine dehydroxylase (Dadh) to m-tyramine.

The intestinal catecholamine environment is also shaped by the gut microbiota. Several taxa metabolize L-DOPA and dopamine, thereby competing with host pathways [9]. A well-defined interspecies route involves *Enterococcus faecalis* converting L-DOPA to dopamine via a pyridoxal-5′-phosphate–dependent TyrDC, followed by *Eggerthella lenta* converting dopamine to *m*-tyramine via a molybdenum-dependent Dadh (Figure 1). Because carbidopa does not inhibit bacterial TyrDC, microbial metabolism may contribute to variability in L-DOPA bioavailability and supports the rationale for microbial enzyme–specific inhibitors [20].

Functionally, intestinal dopamine exerts context-dependent effects on motility and secretion and contributes to barrier integrity and immune tone. Receptor subtype distribution (D1–D5) across enteric neurons, smooth muscle, epithelia, and immune cells likely explains divergent findings across experimental systems [6,21,22,23,24]. Clinically, altered enteric dopamine signaling is associated with dysmotility (notably constipation) and may interact with microbiota-driven presystemic L-DOPA metabolism to influence treatment variability in PD [25,26,27].

A key distinction is that host dopamine production is tightly regulated, whereas microbial decarboxylation of luminal L-DOPA can increase local dopamine exposure and alter dopaminergic pharmacokinetics [28,29,30]. HPLC-based studies and germ-free or antibiotic-treated models demonstrate microbiota-dependent shifts in intestinal dopamine levels, although the quantitative contribution of microbial versus host sources in humans remains incompletely defined [31,32,33]. Beyond motility and secretion, dopaminergic signaling modulates mucosal immunity via dopamine receptors on T cells and antigen-presenting cells, linking microbiota-driven dopaminergic changes to intestinal and systemic immune phenotypes [18,34,35,36].

Taken together, intestinal dopamine emerges as a central integrative signal within the MGBA. Delineating endogenous sources, microbial metabolism, receptor-specific mechanisms, and clinical implications provides a mechanistic framework for subsequent discussion of microbial dopamine metabolism and its relevance to Parkinson’s disease and therapeutic strategies [17,19,37,38,39].

## 4. Microbial Production and Metabolism of Dopamine

The gut microbiota contributes to neurotransmitter biotransformation, with dopamine representing a key example of host–microbe metabolic crosstalk. Although the physiological relevance of microbial dopamine production in vivo remains debated, multiple studies have established bacterial enzymatic pathways involved in catecholamine metabolism (Table 2) [6,39,40].

The best-characterized pathway is the interspecies conversion of L-DOPA. *Enterococcus faecalis* expresses a pyridoxal-5′-phosphate–dependent TyrDC that converts L-DOPA to dopamine and is resistant to carbidopa inhibition [8,42]. *Eggerthella lenta* subsequently converts dopamine to *m*-tyramine via Dadh (Figure 1). This sequential metabolism reduces L-DOPA bioavailability and provides a mechanistic basis for between-patient heterogeneity in therapeutic response [11,43].

Additional taxa (e.g., *Lactobacillus*, *Bacillus*, *Clostridium*) have been implicated in catecholamine synthesis or modification, but their quantitative contribution in the human gut remains uncertain due to variability in composition and activity [41]. In vitro cultures can generate dopamine from L-DOPA at millimolar concentrations, and germ-free/antibiotic-treated models show altered intestinal and central dopamine levels relative to colonized controls. HPLC with electrochemical detection remains a reference method, although protocol standardization is limited [44,45,46].

Clinically, enrichment of *Enterococcus* and *Eggerthella* correlates with reduced L-DOPA bioavailability, and *Helicobacter pylori* infection can further impair absorption [47,48,49,50]. Microbiota-targeted strategies under investigation include antibiotics, probiotics, fecal microbiota transplantation, and selective inhibition of bacterial TyrDC/Dadh to complement carbidopa [51,52,53,54]. Collectively, these findings support the gut microbiota as a determinant of dopaminergic homeostasis and dopaminergic pharmacotherapy [55,56,57,58,59]. Importantly, the quantitative contribution of microbial dopamine production to systemic dopaminergic signaling in humans remains incompletely defined.

Microbial dopamine metabolism represents a tangible modifier of host neurochemistry, underscoring the need for integrative approaches combining advanced multi-omics technologies and targeted interventions to enable precision microbiome-based modulation of dopaminergic therapies [60,61,62,63,64,65].

## 5. Microbiota and Levodopa Therapy in Parkinson’s Disease

The efficacy of L-DOPA in PD is shaped by gut microbial metabolism that reduces presystemic availability and contributes to variability in exposure and clinical response (Table 3) [35,66,67]. As detailed in Section 4, the TyrDC–Dadh pathway can divert orally administered L-DOPA into dopamine and *m*-tyramine in a carbidopa-insensitive manner, creating a microbial “metabolic sink” [42,68,69,70].

Clinical observations support these mechanisms. *H. pylori* infection is associated with impaired L-DOPA absorption and motor fluctuations, and eradication can improve motor outcomes. PD-related dysbiosis often features enrichment of *Enterococcus* and *Eggerthella*, correlating with variable motor responses. Cohort data suggest microbiota composition can partially predict differences in L-DOPA effectiveness [71,72].

Interventions aimed at microbial modulation include antibiotics (limited by non-specific effects), probiotics (consistent benefits for constipation and quality of life, mixed motor outcomes), and FMT (pilot safety and preliminary efficacy). Selective inhibitors of bacterial TyrDC and Dadh represent a promising precision strategy by targeting microbial enzymes that escape conventional inhibition [51,73,74].

Translationally, microbial biomarkers (e.g., TyrDC/Dadh gene abundance and catecholamine-derived metabolite profiles) may enable patient stratification and individualized adjunct strategies. Larger multicenter trials integrating microbiome profiling with pharmacokinetics and clinical phenotyping are needed to define efficacy, durability, and safety across populations [75,76,77,78,79].

## 6. Pathways of Communication: From Gut Dopamine to Brain

Gut-derived dopamine can influence systemic and CNS function via four MGBA routes: neural, immune, metabolic/endocrine, and barrier-related mechanisms [80]. Neural signaling is the most direct route; dopamine modulates ENS activity and vagal afferents, and vagotomy abolishes microbiota-driven effects on central dopaminergic circuits in animal models [81,82,83]. Immune signaling provides a second link, as dopamine receptors on immune cells regulate cytokine production (e.g., IL-6, TNF-α, IFN-γ), connecting intestinal dopaminergic changes to peripheral inflammation relevant to PD, MS, and IBD [84,85]. Metabolic and endocrine interactions further integrate dopamine with SCFAs, tryptophan metabolites, and gut hormones (e.g., GLP-1, ghrelin), influencing appetite and reward-related behaviors [86,87]. Finally, dopamine affects epithelial tight junctions, and experimental models associate dopaminergic alterations with changes in intestinal and blood–brain barrier integrity, potentially facilitating neuroinflammatory signaling [88] (Table 4).

**Figure 2:**
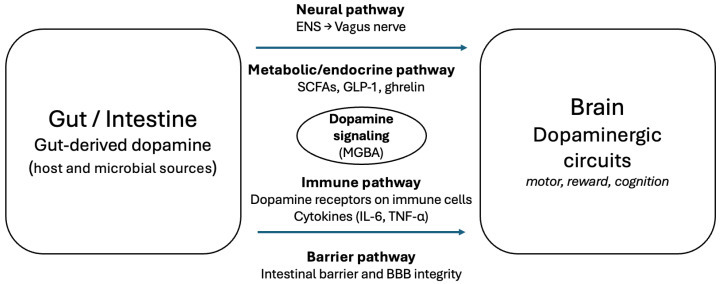
Dopamine-mediated gut–brain axis illustrating neural, metabolic/endocrine, immune, and barrier-related communication pathways influencing brain dopaminergic circuits.

These routes are interdependent, supporting an integrated MGBA model in which dopamine coordinates neural, immune, metabolic, and barrier functions (Figure 2). Clarifying the relative contribution of each pathway may inform targeted interventions, including neuromodulation, dietary strategies, and microbiota-directed approaches to restore barrier and immune homeostasis [89,90].

## 7. Beyond Parkinson’s Disease: Emerging Links

Beyond PD, gut-derived and microbiota-modulated dopamine signaling has been linked to psychiatric, neurodevelopmental, metabolic, and immune-mediated conditions, supporting microbial catecholamine metabolism as a broader host–microbiota interface [91] (Table 5). In depression and anxiety, dysbiosis is associated with altered dopaminergic signaling; germ-free and microbiota-manipulated models show changes in striatal dopamine turnover and behavior, with partial rescue following microbiota transfer. Human studies also report altered microbial composition and catecholamine-related metabolism in major depressive disorder [92,93]. In ASD and ADHD, experimental evidence suggests microbiota modulation can alter dopaminergic metabolism and related social/cognitive behaviors, consistent with a role in neurodevelopmental dopaminergic circuitry [94]. Metabolically, dopamine interacts with SCFAs and appetite-related hormones (e.g., ghrelin, leptin), linking microbial shifts to reward-based feeding, obesity, and hyperphagia in animal models [95]. In IBD, disrupted mucosal dopaminergic signaling may exacerbate inflammation and barrier dysfunction, suggesting microbiota-directed strategies could complement anti-inflammatory approaches [96]. Overall, these associations warrant cross-disciplinary studies and biomarker-driven clinical designs [6,97].

## 8. Therapeutic Perspectives

Therapeutic strategies targeting microbial dopamine metabolism combine microbiota modulation, dietary approaches, selective enzyme inhibition, and precision frameworks, moving toward mechanism-based, microbiome-informed care (Table 6) [98]. Probiotics and prebiotics are the most accessible options; *Lactobacillus*/*Bifidobacterium* formulations improve gastrointestinal symptoms and quality of life in PD, with inconsistent motor effects. Prebiotics and synbiotics may further support beneficial taxa and metabolic outputs relevant to dopaminergic balance [63,99].

Dietary modulation provides a complementary, non-invasive approach. High-fiber diets increase SCFA production, which can influence dopaminergic signaling, while polyphenol-rich diets may inhibit microbial decarboxylase activity involved in L-DOPA degradation [100,101]. FMT can more directly restructure microbial communities; pilot PD studies suggest safety and preliminary efficacy but require standardized, adequately powered trials before routine implementation [64,76].

Pharmacological advances are particularly promising. Carbidopa inhibits host AADC but does not block microbial TyrDC; selective inhibitors targeting bacterial TyrDC and Dadh enhance L-DOPA bioavailability in preclinical models and support dual-inhibition strategies [54,102]. Precision approaches integrating microbiome sequencing, metabolomics, and pharmacokinetics may enable stratified adjuncts (diet/probiotics/enzyme inhibition) aligned with individual microbial and metabolic profiles [103,104]. Future paradigms will likely be combinatorial, with long-term safety evaluation—especially for FMT and novel inhibitors—remaining essential [105].

## 9. Conclusions and Future Directions

Dopamine–microbiota interactions represent a rapidly evolving interface spanning neurogastroenterology, microbiology, metabolism, and clinical neuroscience. Host- and microbiota-derived dopamine shape gastrointestinal motility, secretion, immune regulation, and barrier integrity, while MGBA signaling routes link intestinal dopaminergic changes to brain-relevant physiology. Mechanistic characterization of microbial TyrDC and Dadh has refined the understanding of L-DOPA pharmacokinetics and provides a plausible basis for heterogeneity in therapeutic response in PD. Beyond PD, emerging evidence implicates dopamine–microbiota crosstalk in mood disorders, neurodevelopmental conditions, metabolic dysfunction, and immune-mediated disease.

Key gaps include defining the quantitative contribution of microbial dopamine metabolism in humans, improving assay standardization for dopamine-related metabolites, and establishing long-term safety and efficacy for microbiota-targeted interventions (including FMT and microbial enzyme inhibitors). Inter-individual variability driven by diet, medications, genetics, and geography further complicates translation across cohorts.

Future research priorities include (i) biomarker development (microbial enzyme abundance and metabolomic signatures); (ii) integrated multi-omics to connect genes, transcripts, and metabolites to phenotypes; (iii) multicenter clinical trials combining microbiome profiling with pharmacokinetics and clinical endpoints; and (iv) precision strategies aligning dopaminergic therapy with individual microbial and metabolic profiles. Together, these efforts may optimize dopaminergic therapies and expand microbiome-informed interventions across neurological, metabolic, and immune-related disorders.

## Figures and Tables

**Table 1:** Sources, mechanisms, and local functions of intestinal dopamine.

Source of Intestinal Dopamine	Mechanism	Principal Local Functions	Evidence (Representative References)
Enterochromaffin (EC) cells	Tyrosine → L-DOPA → dopamine via aromatic L-amino acid decarboxylase (AADC); paracrine release to ENS	Modulation of motility, secretion, epithelial barrier tone	MGBA overviews; *Front. Microbiol.* 2025 [1]; *Metabolites* 2024 [17]
Enteric nervous system (ENS) and sympathetic fibers	Neuronal synthesis and synaptic release onto smooth muscle and secretory epithelium	Fine-tuning of peristalsis (D1/D2-family effects), fluid and electrolyte transport	Reviews on gut dopaminergic signaling; *J. Cell. Physiol.* 2017 [18]
Microbiota-derived pathways	(i) *Enterococcus faecalis* TyrDC: L-DOPA → dopamine; (ii) *Eggerthella lenta* Dadh: dopamine → *m*-tyramine	Potential alteration of luminal catecholamine exposure; reduces L-DOPA availability	*Science* 2019 [8]; *eLife* 2020 [9]; *Nat. Commun.* 2019 [10]
Additional microbial taxa	Reported dopamine synthesis by *Lactobacillus*, *Bacillus*, *Clostridium* spp.	Potential neuromodulation; hypothesized barrier and immune effects	*Biomedicines* 2022 [6]
Immune compartment cross-talk	Dopamine receptors on T cells and macrophages; cytokine modulation	Regulation of mucosal immunity and inflammation	*Brain* 2021 [11]; *Cell* 2016 [19]

**Table 2:** Key microbial enzymes involved in dopamine metabolism.

Microbial Species	Enzyme	Substrate → Product	Key References
*Enterococcus faecalis*	Tyrosine decarboxylase (TyrDC), PLP-dependent	L-DOPA → dopamine	Rekdal et al., *Science* 2019 [8]
*Eggerthella lenta*	Catechol dehydroxylase (Dadh), molybdenum-dependent	Dopamine → *m*-tyramine	Bisanz et al., *Drug Metab. Dispos.* 2018 [38]
*Clostridium* spp.	Multiple decarboxylases and reductases	Tyrosine/catecholamines → various metabolites	Strandwitz, *Brain Res.* 2018 [4]
*Lactobacillus* spp., *Bacillus* spp.	Putative tyrosine decarboxylases	Tyrosine → dopamine	Lyte, *BioEssays* 2011 [15]
*Helicobacter pylori*	Indirect effects on absorption and metabolism	Reduced bioavailability of therapeutic L-DOPA	*Front. Neurol.* 2023 [41]

**Table 3:** Key clinical evidence linking microbiota to L-DOPA therapy in Parkinson’s disease.

Study/Period	Intervention/Population	Main Findings	Reference
2010s, observational cohorts	*H. pylori* eradication in PD patients	Improved L-DOPA absorption and motor symptoms	*Brain* 2021 [11]
Rekdal et al. 2019; Bisanz et al. 2018	Mechanistic characterization of TyrDC (*E. faecalis*) and Dadh (*E. lenta*)	Defined two-step microbial L-DOPA degradation pathway	*Science* 2019 [8]; *Drug Metab. Dispos.* 2018 [38]
2022–2024 pilot studies	Fecal microbiota transplantation (FMT) in PD	Safe; preliminary benefit for motor and non-motor symptoms	*Front. Neurol.* 2023; ClinicalTrials.gov [48]
2024–2025 experimental therapies	Selective bacterial TyrDC/Dadh inhibitors + carbidopa	Enhanced systemic and central L-DOPA availability (preclinical/early translational)	*Eur. J. Pharm. Sci.* 2025 [12]

**Table 4:** Major microbiota–gut–brain axis communication pathways involving dopamine.

Pathway	Mechanism	Representative Evidence	Implications
Neural (ENS and vagus nerve)	Dopamine modulates enteric neurons and vagal afferents; vagotomy abolishes microbial effects	Germ-free and vagotomy animal models	Links gut dopamine to central motor and reward circuits
Immune	Dopamine receptors on T cells and macrophages regulate IL-6, TNF-α, IFN-γ	PD and IBD models	Peripheral immune modulation influences neuroinflammation
Metabolic/Endocrine	Interaction with SCFAs, tryptophan metabolites, GLP-1, and ghrelin	Metabolomics and multi-omics MGBA studies	Regulation of appetite, energy balance, reward
Barrier function (gut and BBB)	Dopamine modulates tight junction proteins and permeability	Experimental models	Facilitates cytokine/metabolite entry into CNS

**Table 5:** Conditions associated with altered microbial dopamine signaling.

Condition	Proposed Mechanism	Key Evidence	Therapeutic Implications
Depression and anxiety	Microbial modulation of dopaminergic mood and reward circuits	Germ-free models; human dysbiosis studies	Potential adjunctive probiotic or FMT strategies
Neurodevelopmental disorders (ASD, ADHD)	Microbiota-driven alterations in striatal dopamine signaling	Animal models	Microbiome-targeted adjunct therapies
Metabolic disorders and feeding behavior	Interaction with SCFAs, ghrelin, leptin affecting reward-based eating	Animal studies	Targeting dopaminergic pathways for weight management
Gastrointestinal and immune disorders (IBD)	Dopamine-dependent regulation of mucosal immunity and barrier integrity	Human mucosal studies	Probiotic/prebiotic strategies to reduce inflammation

**Table 6:** Therapeutic strategies targeting microbial dopamine metabolism.

Strategy	Mechanism	Stage of Evidence	Representative References
Probiotics/Prebiotics	Modulate microbial composition; enhance beneficial taxa (*Lactobacillus*, *Bifidobacterium*)	Pilot RCTs; animal models	*Brain* 2021 [11]; *Neurology* 2021 [46]
Dietary interventions	High-fiber diet → SCFA production; polyphenols inhibit microbial decarboxylases	Observational and experimental studies	*Metabolites* 2024 [17]
Fecal microbiota transplantation (FMT)	Restores microbial balance; indirectly normalizes dopamine metabolism	Pilot clinical trials in PD	*Front. Neurol.* 2023 [48]
Pharmacological inhibition	Small-molecule inhibitors of bacterial TyrDC and Dadh	Preclinical and translational studies	*Science* 2019 [8]; *eLife* 2020 [9]
Precision medicine approaches	Biomarker-guided stratification (TyrDC/Dadh genes, *m*-tyramine, metabolomics)		

## Data Availability

No new data were created or analyzed in this study. Data sharing is not applicable to this article.

## Abbreviations

The following abbreviations are used in this manuscript:

MGBA	microbiota–gut–brain axis
TyrDC	tyrosine decarboxylase
PD	Parkinson’s disease
GABA	gamma-aminobutyric acid
CNS	central nervous system
GI	gastrointestinal
ASD	autism spectrum disorder
ADHD	attention-deficit/hyperactivity disorder
ENS	enteric nervous system
EC	enterochromaffin cells
FMT	fecal microbiota transplantation
